# MHC Class II Polymorphisms, Autoreactive T-Cells, and Autoimmunity

**DOI:** 10.3389/fimmu.2013.00321

**Published:** 2013-10-10

**Authors:** Sue Tsai, Pere Santamaria

**Affiliations:** ^1^Department of Microbiology, Immunology and Infectious Diseases, Faculty of Medicine, Julia McFarlane Diabetes Research Centre, Snyder Institute for Chronic Diseases, University of Calgary, Calgary, AB, Canada; ^2^Institut D’Investigacions Biomediques August Pi i Sunyer, Barcelona, Spain

**Keywords:** MHC class II, autoimmune diseases, susceptibility genes, resistance genes, type 1 diabetes, autoreactive T cells, T regulatory cells

## Abstract

Major histocompatibility complex (MHC) genes, also known as human leukocyte antigen genes (HLA) in humans, are the prevailing contributors of genetic susceptibility to autoimmune diseases such as Type 1 Diabetes (T1D), multiple sclerosis, and rheumatoid arthritis, among others (1–3). Although the pathways through which MHC molecules afford autoimmune risk or resistance remain to be fully mapped out, it is generally accepted that they do so by shaping the central and peripheral T-cell repertoires of the host toward autoimmune proclivity or resistance, respectively. Disease-predisposing MHC alleles would both spare autoreactive thymocytes from central tolerance and bias their development toward a pathogenic phenotype. Protective MHC alleles, on the other hand, would promote central deletion of autoreactive thymocytes and skew their development toward non-pathogenic phenotypes. This interpretation of the data is at odds with two other observations: that in MHC-heterozygous individuals, resistance is dominant over susceptibility; and that it is difficult to understand how deletion of one or a few clonal autoreactive T-cell types would suffice to curb autoimmune responses driven by hundreds if not thousands of autoreactive T-cell specificities. This review provides an update on current advances in our understanding of the mechanisms underlying MHC class II-associated autoimmune disease susceptibility and/or resistance and attempts to reconcile these seemingly opposing concepts.

## Introduction

Major histocompatibility complex (MHC) class II molecules are surface heterodimers expressed by thymic epithelial cells and professional antigen-presenting cells (APCs) that present antigenic peptides to T-cell receptors (TCR) on cognate T-cells. A developing thymocyte first encounters a highly heterogeneous array of endogenous (self) peptide-MHC (pMHC) complexes on thymic APCs. Combinations of self-peptides and MHC molecules instruct thymocytes to either survive or perish, based on the affinity/avidity with which their TCRs bind pMHC. Through processes referred to as thymic positive and negative selection, thymocytes that bind self pMHCs with either intermediate-to-low or high avidity are instructed to survive or perish, respectively (4). As a result, the peripheral T-cell repertoire is largely composed of T-cells capable of recognizing foreign peptides in the context of self MHCs or self pMHCs that are expressed exclusively in peripheral tissues. This forms the basis of self vs. non-self discrimination in adaptive immunity, ensuring that the peripheral immune system is populated by a diverse repertoire of T-cells capable of mounting immune responses against an essentially limitless universe of foreign antigens, while minimizing the risk of causing autoimmunity. Nonetheless, thymic negative selection is not an “air-tight” process and, as a result, some autoreactive specificities evade negative selection and populate the periphery (5, 6). As they possess the potential to cause autoimmunity, additional regulatory mechanisms have evolved to keep them in check, such as the induction of anergy or activation-induced cell death by tolerogenic APCs (7, 8), or suppression by regulatory T-cells (Tregs). The latter can dampen autoimmune responses through several mechanisms, including direct inhibition of APCs (9–14).

## MHC Polymorphisms and Autoimmunity

Antigenic peptides are embedded in the peptide-binding groove of the MHC class II molecule, which consists of two flanking alpha helices atop a beta-pleated sheet. With some exceptions, these peptides are anchored onto the MHC class II binding cleft through their amino acid side chains at four positions, termed pockets 1, 4, 6, and 9. Interestingly, autoimmune disease-promoting MHC class II alleles often differ from disease-non-promoting ones by only a few amino acids that are primarily located at the TCR-MHC interface or in the peptide-binding groove, at times adjacent to key anchoring pockets (15, 16). This has suggested that MHC-linked disease risk is associated with differences in the repertoire of self-peptides that are presented to T-cells. Indeed, substantial evidence from different autoimmune diseases supports this possibility. For instance, HLA-DRB1 alleles affording susceptibility to Rheumatoid arthritis (RA) share a conserved sequence of amino acids at residues 67–74 of the DRβ chain (17), which is situated on one of the alpha helices flanking the peptide-binding groove. Genome-wide SNP analyses of seropositive RA patents vs. healthy controls confirmed the genetic contribution of two of these residues, 71 and 74, as well as three others, all of them located in the peptide-binding groove, to RA susceptibility (18). Available peptide-binding data from RA-predisposing DRB1*0401 and *0404 vs. RA-protective *0402 alleles revealed an association between the repertoire of bound peptides and polymorphisms at position 71 (19), although it remains to be determined how these associations contribute to autoreactivity. Of note, DRB1 residues 71 and 74 appear to be a hotspot for other autoimmune disease-associated polymorphisms as well, including autoimmune thyroiditis (20) and Multiple Sclerosis (MS) (21), owing to their vicinity to the P4 pocket.

A similar phenomenon may underlie the MHC-T1D associations. By comparing disease-promoting and -protective class II molecules in mice and humans, it was found that polymorphisms at DQβ chain positions 56 and 57, located on one of the two alpha helices flanking the peptide-binding groove, strongly correlated with both disease susceptibility and resistance (22, 23). Alleles that encode Asp at position 57 in humans (or Pro56 and Asp57 in mice) afford protection, whereas those that encode Ser or Ala (or His56 and Ser57 in mice) at these positions afford risk (22, 23). In fact, an alignment of protective murine I-Aβ chains with their diabetogenic I-Aβ^g7^ counterpart reveals a striking consensus sequence spanning residues 56–67. The I-Aβ^g7^ His56 and Ser57 residues, unlike the disease-protective Pro56 and Asp57 residues, fail to form a salt bridge and neutralize the positive charge imparted by Arg76 on the I-Aα^d^ chain, resulting in a positively charged P9 pocket that favors the binding peptides carrying acidic amino acids at the C-terminus (24, 25). This positive charge results in the unstable binding of many self-peptides to I-A^g7^ and presumably the presentation of a narrower range of epitopes (15, 26–30). Similarly in humans, the predisposing class II variants, DQ2 and DQ8, carry an Ala at the beta chain position 57 and exhibit preference for binding peptides carrying acidic residues at their carboxyterminus (31, 32).

## MHC Polymorphism and Positive/Negative Selection

**Figure 1:**
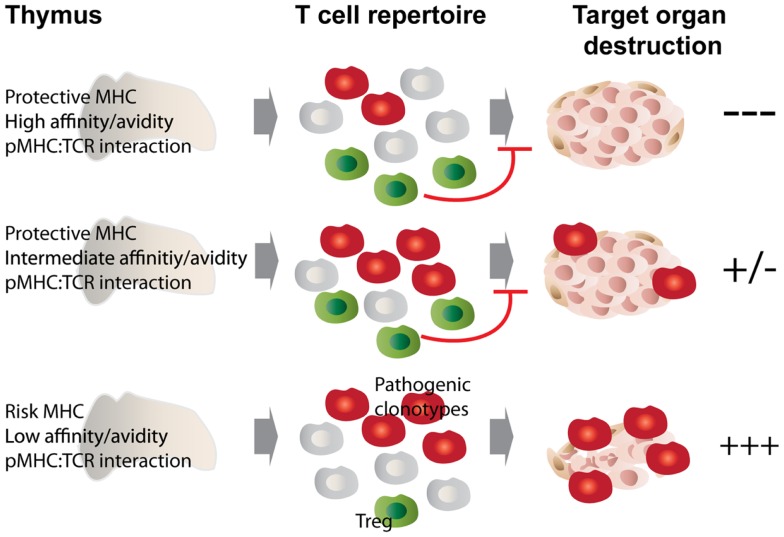
**MHC class II polymorphisms afford autoimmune disease resistance through shaping the T cell and Treg repertoire**. MHC class II molecules that afford disease risk allow the escape of pathogenic autoreactive T cells from central tolerance, while protective MHC class II molecules confer disease resistance through promoting negative selection as well as autoreactive Treg development. We propose that these processes are governed by the affinity/avidity with which pMHCs are bound by TCRs. Disease-protective pMHC interact with MHC-promiscuous, autoreactive thymocytes with increased affinity/avidity (top and middle panels), leading to enhanced negative selection and agonist selection of Tregs, which then dampen the autoimmune response through various mechanisms. In the bottom scenario, low affinity/avidity interaction between pMHC and autoreactive TCRs leads to defective negative selection and Treg development, with the net result of autoimmunity.

A direct consequence of altered peptide presentation is the undermining of a number of MHC class II-mediated processes. For example, MHC class II molecules that afford autoimmune susceptibility would promote positive selection of pathogenic autoreactive thymocytes in the cortex while failing to trigger their subsequent deletion in the medulla. On the other hand, protective class II would select a repertoire of T-cells endowed with decreased pathogenicity (33). Another process that likely bears the brunt of such alteration is negative selection; this is supported by observations made in animal studies comparing disease-promoting MHCs to their disease-protective counterparts differing by only a few amino acids at positions located in the peptide-binding groove. In these studies, protective class II molecules curbed autoimmunity by promoting the negative selection of certain, in this case class II-promiscuous, autoreactive T-cell specificities (34–38). The latter observations brought forth the idea that protective class II polymorphisms located at or near the peptide-binding groove may be recognized by certain MHC-promiscuous autoreactive TCRs with an avidity and/or affinity above the threshold required for negative selection. We have recently shown that dendritic cells play a key role in this process (38, 39). Figure 1 depicts the proposed relationship between pMHC:TCR interaction strength and its outcome in terms of the selection of pathogenic vs. regulatory T cell clonotypes, and how MHC polymorphisms play into this selective process.

These observations are compatible with the idea that low-avidity presentation of self-peptides by disease-promoting MHCs to potentially pathogenic autoreactive thymocytes would contribute to autoimmune disease susceptibility by impairing their negative selection without compromising positive selection, as the affinity/avidity thresholds for these processes are different (40). Exposure to increased antigenic loads and/or differentially processed epitopes in the periphery (41) could then suffice to fuel the activation of these thymic escapees and their recruitment into an autoimmune response. Our studies with the diabetogenic, MHC-promiscuous (but I-A^g7^-restricted) 4.1-TCR are entirely compatible with this view (34–36, 38, 39).

Nevertheless, not all protective class II molecules are equally effective at eliciting central tolerance of diabetogenic TCRs (33, 36, 38, 39, 42), indicating that class II-associated resistance to autoimmunity cannot be solely accounted for by this process (deletion of key autoreactive T-cell specificities). Furthermore, it is difficult to understand how deletion of one or a handful of autoreactive T-cell types might be able to blunt autoimmune responses involving many other specificities. One could argue that enhanced negative selection of a key immunodominant specificity playing a critical role in disease initiation would be sufficient. However, without knowing if a given specificity is capable of singularly initiating disease or swaying its outcome, the consequences of its deletion are hard to predict. Most likely, deleting any single or combination of T-cell specificity(ies) will not alter the course of a disease that is mediated by a polyclonal T-cell repertoire, as we have previously demonstrated (43).

## MHC Polymorphism and Treg Development

It is therefore likely that enhanced central tolerance of autoreactive T-cells by protective class II molecules cannot be the only mechanism underlying the MHC-associated autoimmune disease resistance. Engagement of pMHCs expressed on thymic medullary epithelial cells or bone marrow-derived APCs by thymocytes can yield different outcomes, depending on the avidity of the interaction. Although the most avid pMHC:TCR interactions invariably promote thymocyte apoptosis, strong interactions that fall below the threshold for deletion can result in an alternative outcome – the generation of Treg cells. This process, also referred to as “agonist selection” of regulatory T-cells (44), occurs within a window of affinity/avidity that spans the thresholds of positive and negative selection (45).

Since many of the early mechanistic studies on this topic predated the discovery of Treg cells by as long as a decade (46), the potential effects of autoimmune disease-associated class II polymorphisms on Treg cell development and/or function were not investigated in detail. However, some of these early studies showed that protective MHC class II molecules induced dominant, T-cell-transferrable tolerance (47, 48).

**Figure 2:**
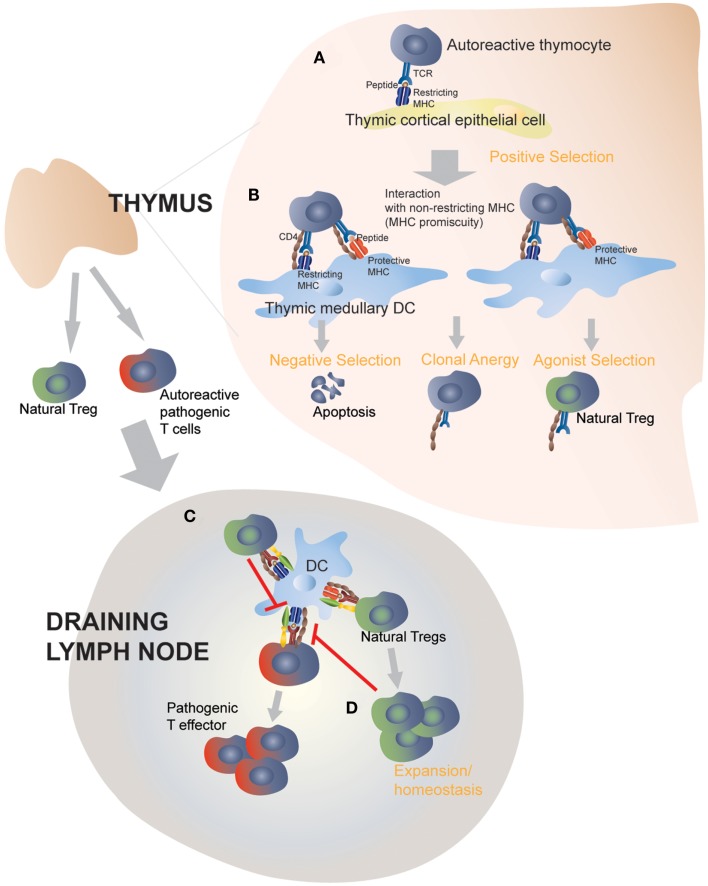
**Protective MHC class II molecules mediate central and peripheral tolerance by targeting MHC-promiscuous autoreactive TCRs**. Positive selection on I-A^g7^ in the thymic cortex determines the MHC restriction of thymocytes **(A)**. In the absence of protective MHC molecules, negative selection is defective and fail to purge the repertoire of pathogenic autoreactive thymocytes (not shown) (**B**). Transgenic expression of disease-protective MHC class II molecules on dendritic cells leads to enhanced negative selection and clonal anergy of autoreactive, MHC-promiscuous thymocytes, and promotes autoreactive Treg differentiation and functional development (**C**). Thymic derived Tregs then exit into the periphery and suppress the activation of pathogenic T cells by directly acting on autoantigen-loaded APCs. This step does not require protective MHC class II molecules, although a role of protective MHC class II molecules, expressed on peripheral APCs, in perpetuating autoreactive Tregs or enhancing their homeostasis cannot be ruled out **(D)**.

The observation that certain (albeit not all) protective class II molecules lost their anti-diabetogenic effects in monoclonal (RAG-deficient) autoreactive TCR-transgenic mice led us to suspect a contribution by endogenous (non-TCR-transgenic) autoreactive T-cells to this process (38, 39). We measured the frequency and function of Treg cells in TCR-transgenic and non-transgenic NOD mice co-expressing T1D-protective class II alleles (38, 39). Autoreactive TCR-transgenic NOD mice co-expressing anti-diabetogenic class II alleles harbored increased numbers of autoreactive Treg cells that had superior regulatory activity as compared to Treg cells arising in mice only expressing wild-type H-2^g7^. In non-TCR-transgenic animals expressing protective class II alleles, increases in Treg numbers were restricted to the target organ and local draining lymph nodes, where there was an enrichment for autoreactive specificities. These observations suggested that protective class II molecules promote the differentiation of potentially pathogenic autoreactive thymocytes into Treg cells, which would then be able to effectively blunt all other autoreactive T-cell responses by suppressing autoantigen presentation in the draining lymph nodes (38, 39). Furthermore, consistent with the model of agonist selection, such increases in MHC class II-induced autoreactive Treg selection coincided with the up-regulation of CD5, CD69, and Nur77 on thymocytes, which are induced by high affinity pMHC:T-cell interactions (49), and were paralleled by negative selection (38). Expression of sub-tolerogenic, but still anti-diabetogenic class II variants in these autoreactive TCR-transgenic NOD mice promoted increases in the *in vivo* regulatory capacity of the peripheral Treg cell pool, owing to increases in the peripheral frequency of autoreactive regulatory T-cell specificities, further substantiating the above observations (39). Remarkably, all these effects were driven by protective class II types expressed exclusively in DCs, therefore suggesting that class II-driven autoreactive Treg formation occur in the thymic medulla (38, 39). Figure 2 highlights the proposed interactions between disease-promoting and protective MHC class II molecules on thymic APCs and autoreactive TCRs on thymocytes. The nature of such interaction will be further discussed below.

These observations help us understand how a single class II molecule can blunt an autoantigenically complex T-cell response: agonist-induced development of autoreactive Tregs from thymocytes expressing MHC-promiscuous autoreactive TCRs (Figures 1 and 2). Still, several questions remain as to how protective class II molecules manage to do so. What are the changes in the bound peptide repertoire, and/or the overall pMHC structure that promote enhanced Treg selection? Studies using H-2DM-deficient mice predicted that such a process is peptide-dependent but not necessarily peptide-specific, suggesting a dominant role for MHC residues, as opposed to peptide residues, in driving this outcome (35). Another observation that provides useful insights is the requirement for endogenous TCRs in protective MHC-induced autoreactive Treg formation. Looking at the three different autoreactive TCR/MHC class II combinations that were explored, we can conclude that not all autoreactive TCRs are overtly MHC-promiscuous or promiscuous for all protective MHC types and therefore capable of engaging protective MHC (38, 39). Instead, we hypothesize that the endogenous autoreactive thymocyte repertoire (positively selected and restricted by disease-promoting MHC types) is inherently promiscuous for other (suppressing or even non-disease-promoting) MHC types, and that only those TCRs that can engage the latter with an affinity/avidity above the threshold required for agonist-induced Treg formation will contribute to disease suppression. In other words, MHC alleles affording dominant resistance to a given autoimmune disease are those capable of harnessing the intrinsic MHC promiscuity of pathogenic TCRs to generate autoreactive Treg cells.

Since class II molecules are expressed on all professional APCs, one has to wonder if protective class II types also contribute to autoimmune disease suppression by shaping the post-thymic peripheral T-cell repertoire. It is well accepted that disease-predisposing class II molecules are responsible for presenting self epitopes to autoreactive T-cells, leading to their activation and therefore exerting a direct impact on the effector phase of the autoimmune response. Through antigen presentation, predisposing class II molecules have also been proposed to contribute to autoimmune inflammation by modulating the cytokine milieu produced by T-cells (50). The contribution of disease-protective class II in the periphery, however, is less clear. Certain studies implicated protective class II molecules in mechanisms such as “determinant capture” that would interfere with or compete against self-antigen presentation by disease-predisposing MHCs (51–53). These mechanisms, although plausible, were countered by other reports (54–56). Although we find that autoreactive Treg cells need not have to engage protective pMHC class II complexes in the periphery to effect disease suppression (38, 39), it remains to be determined whether protective MHC class II play a role in their homeostatic survival. Repeated encounters with disease-promoting pMHC in the periphery (57, 58) may be sufficient for both survival and suppression.

## How Autoreactive TCRs Bind Protective vs. Disease-Promoting MHCs

The above interpretation of the data implies that certain autoreactive T-cell specificities have the capacity to recognize more than one peptide in the context of more than one type of class II molecule (Figure 2). In fact, it has been established that individual TCRs have the potential to recognize a surprisingly wide array of peptides in the context of a single MHC molecule (59), or multiple MHC molecules presenting one or more peptide(s) (60, 61). Furthermore, we and others have provided evidence that pathogenic autoreactive TCRs are inherently “MHC-promiscuous” (34, 62–64). In this context, MHC diversity, we propose, can be viewed as nature’s guard against T-cell-driven autoimmunity. By exploiting their tendency to “stick” to certain class II molecules, protective MHC types can either eliminate autoreactive T-cells at the get-go and/or, most importantly, instruct them to join the ranks of regulatory T-cells. The molecular basis underlying the interaction between autoreactive TCRs and protective MHCs remains as yet undefined. Structural studies of autoreactive TCRs cloned from relapsing-remitting MS patients or animals induced to develop a similar demyelinating disease (EAE) revealed anomalous characteristics of pMHC binding by these autoreactive TCRs, which engage cognate peptide in the context of disease-predisposing class II molecules in an atypical manner that is distinct from the binding topology of conventional TCRs on foreign pMHC complexes (65–67). Altered binding appeared to be associated with fewer contacts between the TCR and the peptide. In some cases, the TCR/pMHC interaction leaned toward the N-terminal end of the bound peptide, and in others the TCR/pMHC contact was weakened by the altered peptide docking on the MHC, due to the partial occupancy of the peptide-binding groove (65). Whether a pathogenic autoreactive TCR behaves differently in the presence of a disease-protective MHC remains to be determined. As we inferred from functional studies, protective MHC molecules may be recognized by a given autoreactive TCR with higher affinity/avidity than a disease-promoting MHC type, thereby correcting deficiencies in negative selection or Treg development. We propose that disease-protective class II molecules signal the deletion of autoreactive thymocytes as well as their development into Tregs by taking advantage of the atypical binding characteristics of certain autoreactive TCRs, i.e., their intrinsic MHC promiscuity.

## Concluding Remarks

The implications of MHC polymorphism evolution vis-à-vis health and disease are manifold. Polymorphic class I and II molecules endow the adaptive immune system with the flexibility to recognize a wide array of pathogens, in terms of both selecting a diverse T-cell repertoire in the thymus, and enabling their activation in the periphery through antigen presentation. In close association to their function in generating TCR diversity through thymic education, MHC molecules also have the important role of discouraging T-cells from brewing autoimmunity. MHC polymorphisms affect the outcome of this educational process, with protective MHC purging harmful clonotypes and disease-promoting MHC permitting their escape. Furthermore, a previously unappreciated role of protective class II molecules in autoimmune disease resistance has been identified: mobilization of self-reactive Treg cells to fight harmful self-reactivity. We propose that protective class II molecules do so by engaging MHC-promiscuous, autoreactive thymocytes in a manner that promotes Treg formation, and that future work on elucidating the interaction between autoreactive TCRs and protective class II molecules will shed light on this very important issue.

## Conflict of Interest Statement

The authors declare that the research was conducted in the absence of any commercial or financial relationships that could be construed as a potential conflict of interest.
